# Supplementary material for: Ego-Resiliency, Life Satisfaction and Symptoms of Anxiety and Depression Among Students of Pro-Health Faculties During COVID-19 Pandemic
Source: Healthcare (Basel). 2025 Apr 27;13(9):1008. doi: 10.3390/healthcare13091008 (PMC12072020; doi:10.3390/healthcare13091008)
Supplement: Supplementary file 1 [file healthcare-13-01008-s001.zip › healthcare-3549712-supplementary.pdf]

Supplementary material: Various differences between medical students and students at Academy of Physical Education (APhE).

Variable	Medical students			
	**ER**	**SWLS**	**ANXIETY**	**DEPRESSION**
ER	1.000	0.518	-0.396	-0.443
SWLS	0.518	1.000	-0.507	-0.603
ANXIETY	-0.396	-0.507	1.000	0.638
DEPRESSION	-0.443	-0.603	0.638	1.000

Variable	Physical education students (APhE)			
	**ER**	**SWLS**	**ANXIETY**	**DEPRESSION**
ER	1.000	0.436	-0.374	-0.426
SWLS	0.436	1.000	-0.441	-0.526
ANXIETY	-0.374	-0.441	1.000	0.636
DEPRESSION	-0.426	-0.526	0.636	1.000

Variable	Male probants			
	**ER**	**SWLS**	**ANXIETY**	**DEPRESSION**
ER	1.000	0.392	-0.348	-0.427
SWLS	0.392	1.000	-0.499	-0.533
ANXIETY	-0.348	-0.499	1.000	0.619
DEPRESSION	-0.427	-0.533	0.619	1.000

Variable	Women probants			
	**ER**	**SWLS**	**ANXIETY**	**DEPRESSION**
ER	1.000	0.511	-0.412	-0.443
SWLS	0.511	1.000	-0.481	-0.595
ANXIETY	-0.412	-0.481	1.000	0.671
DEPRESSION	-0.443	-0.595	0.671	1.000

Variable	Marital status - in close relationship			
	**ER**	**SWLS**	**ANXIETY**	**DEPRESSION**
ER	1.000	0.503	-0.488	-0.467
SWLS	0.503	1.000	-0.483	-0.593
ANXIETY	-0.488	-0.483	1.000	0.644
DEPRESSION	-0.467	-0.593	0.644	1.000
